# Profile of Phenolic Compounds of *Prunus armeniaca* L. Leaf Extract Determined by LC-ESI-QTOF-MS/MS and Their Antioxidant, Anti-Diabetic, Anti-Cholinesterase, and Anti-Inflammatory Potency

**DOI:** 10.3390/antiox10121869

**Published:** 2021-11-24

**Authors:** Aneta Wojdyło, Paulina Nowicka

**Affiliations:** Department of Fruit, Vegetable and Nutraceutical Plant Technology, Wrocław University of Environmental and Life Sciences, 37 Chełmońskiego Street, 51-630 Wrocław, Poland; paulina.nowicka@upwr.edu.pl

**Keywords:** online ABTS, ORAC, FRAP, α-amylase, α-glucosidase, pancreatic lipase AChE, BChE, COX

## Abstract

In view of the limited information available in the literature concerning leaves as by-products of *Prunus armeniaca* cultivation, the aim of this work was to identify and characterize their principal polyphenolic constituents by LC-ESI-QTOF-MS/MS and screening in vitro biological potency as antioxidant capacity (ABTS, online ABTS, FRAP, ORAC), antidiabetic (α-amylase, α-glucosidase), anti-obesity (pancreatic lipase), anti-cholinesterase (AChE and BChE), and anti-inflammatory (COX-1 and COX-2) inhibitory activity. Comparison of different polyphenolic extracts of *P. armeniaca* cultivar leaves according to their quantitative composition revealed them to be exceptional sources of hydroxycinnamic acids, and to a lesser extent as sources of flavonols. Polyphenol-rich apricot leaf extract (PrALe) showed the most effective anti-obesity action through inhibition of pancreatic lipase, COX-1 and antioxidant capacity, especially the oxygen radical absorbance capacity, which was particularly correlated with polyphenolic compounds. Online ABTS radical UPLC-PDA-PDA analysis clearly demonstrated that the three predominant compounds of PrALe are quercetin-3-*O*-rutinoside > 5-*O*- and 3-*O*-caffeoylquinic acid, which basically contribute to antioxidant potential. These results assist in the evaluation of plant sources of potential new raw materials for application in different commercial sectors, especially for food, cosmetics and pharmaceuticals production.

## 1. Introduction

The people worldwide are radically changing their views on daily food and creating new categories such as “functional foods”, “superfoods”, and “novel foods”, or focus on the production of new supplements, and nutraceuticals based on natural plant bioactive compounds [1,2]. Fruits and berries are excellent sources of phytochemicals such as various polyphenols, tri- and tetraterpene, and other nutritional substances such as pectins, fibers, minerals, and vitamins. However, the permanent quest for early disease prevention and health promotion inspires a continual search for new natural plant sources with high pro-healthy properties. Owing to the high contents of these compounds, leaves have received additionally higher attention in the last few years as a potential component of a healthy diet. As Ferlemi and Lamari [3] mentioned, leaves are one of the alternative source of bioactive compounds alongside widely consumed fruits and berries. So far, leaves of berries such as rowanberry, crowberry, lingonberry and bilberry have been listed in the Novel Food Catalogue of the European Commission as food supplements. Additionally, leaves today such as sea buckthorn, wild strawberry and blackcurrant are used for herbal tea production and some ingredients of foods. Some leaves have been reported to be used as traditional remedies by different native populations as infusions against asthma, rheumatism, inflammation of the urinary tract, colds or diabetes. Additionally, there are several reports providing information about nutritional and functional properties of extracts of leaves obtained as by-products and used as antioxidant, anti-inflammatory, hypo-cholesterolemic, antidiabetic, anticancer, or antimicrobial agents [3,4,5,6,7,8].

Therefore, there is continually growing interest about the possibility to find new plant materials such as leaves as valuable sources of bioactive compounds. *Prunus armeniaca* L. is a tree represented the Rosaceae family whose native origin is Central and western Asia including Armenia and China and is today cultivated in many subtropical and tropical areas of the world [9]. The total world production of *P. armeniaca* in recent years was up to 4.1 million metric tons [10], and is still growing. Turkey, Iran, Pakistan, Uzbekistan, and Algeria are leading production countries with high global exports and consumption. *P. armeniaca* fruits are characterized by high nutritional value, rich in carotenoids, fiber, minerals, and organic acids, and they are utilized in the food industry for the production of canned and dried fruits, jams, purees, frozen (79%), and for fresh consumption (20%) [11]. After their basic industrial processing, a large amount of agro-industrial by-products is generated. The fibrous stem of *P. armeniaca* is used for animal bedding, biocomposites, absorbent materials, textiles, paper and pulp production, insulation mats, medium density fiberboards, etc. Other by-products such as seeds and bark have some medicinal purposes, but still only leaves are agro-utilized with few or no further applications. However, they could be a rich source of bioactive compounds, which can be applied as ingredients for pharmaceutical or functional food products. Owing to the high contents of bioactive compounds, leaves have received more attention in the last few years as potential new plant sources of these components for the food and pharmaceutical industry [12,13,14,15,16,17]. Additionally, consumers prefer and accept the use of natural food ingredients as additives because of their safety and availability.

Due to the limited information available in the literature about leaves as by-products of *P. armeniaca* cultivation, the aim of this work was to identify and characterize their principal polyphenolic constituents by LC-ESI-QTOF-MS/MS and screening in vitro biological effects such as antioxidant capacity (ABTS, online ABTS, FRAP, ORAC), antidiabetic (α-amylase, α-glucosidase), antiobesity (pancreatic lipase), anti-cholinesterase (AChE and BChE) and anti-inflammatory (COX-1 and COX-2) and inhibitory activity potency. These results will assist in the evaluation of plant sources of materials as potential new raw materials for application in different commercial sectors such as food, cosmetics and pharmaceuticals.

## 2. Materials and Methods

### 2.1. Plant Materials

Somo, Harcot, and Early orange—three cultivars of *Prunus armeniaca* L.—leaves were collected on May 2021 from different parts (upper and lower) of 3 selected trees (grown by spacing 2 × 3 m) without special fertilization and irrigation located at the Research Station for Cultivar Testing in Zybiszów, Poland. Leaves (1000 ± 10 g) were cut, rapidly frozen by nitrogen gas and processed to freeze-dried for 24 h (Alpha 1-4 LSC freeze-dryer; Martin Christ GmbH, Osterode am Harz, Germany) and finally were milled to powder with a laboratory mill (IKA 11A; Staufen, Germany).

### 2.2. Preparing Polyphenol-Rich Apricot Leaf Extract (PrALe)

To obtain an extract rich in polyphenols of *P. armeniaca* L. leaf powder after freeze-drying was mixed with 50% ethanol, sonicated for 20 min (Sonic 6D; Polsonic, Warsaw, Poland), occasionally mixed, and supernatant was separated by centrifuged for 10 min at 19,000× *g* (MPW-55; Warsaw, Poland). The residue was reextracted 3–4 times and all obtained fractions were mixed and ethanol was evaporated at 40 °C by a scale rotary evaporator (Hei-VAP Expert, Heidolph; Schwabach, Germany). The obtained aqueous fraction was loaded into a glass column (50 cm; Ø 6 cm) filled with Amberlite polymeric XAD-16 resin previously washed with water. The absorbed aqueous fraction was washed with water (1 mL/min) to remove sugars, organic acids or other ballast substances and then polyphenolic compounds were eluted with ethanol (80 and 100%; 1 mL/min). Collected fractions were mixed and, after evaporation (Hei-VAP Expert, Heidolph; Schwabach, Germany) of alcohol at 40 °C, the obtained fraction was freeze-dried to obtain the powder of polyphenol-rich apricot leaf extract (PrALe).

### 2.3. Polyphenolic Compounds—Identification and Quantification Analysis

Analysis of polyphenols was carried out on an LC-ESI-QTOF-MS/MS system and Acquity UPLC system equipped with a photodiode (PDA) detector (Waters Corp., Milford, MA, USA). Polymeric procyanidins were measured with an Acquity UPLC system equipped with a fluorescence (FL) detector (Waters Corp., City, Ireland). Chromatographic systems consisted of a binary pump, an autosampler, and a column compartment. The results processed with the Empower 3.0 program (Waters, Milford, MA, USA). The results are presented as mg per kg of dry weight (dw) after analyzing sample three times.

#### 2.3.1. LC-ESI-QTOF-MS/MS System for Analysis of Polyphenolic Compounds

PrALe (0.05 g) was diluted in a mixture water:methanol:ascorbic acid: 37% hydrochloric acid (6.8:3:0.1:0.1; *v*/*v*/*w*/*v*) and was filtered using a 0.45 μm syringe filter (poli(tetrafluoroethylen)membrane) before analysis.

The conditions consisted of a gradient elution using aqueous 0.1% formic acid (*v*/*v*) as mobile phase A and acetonitryl with 0.1% formic acid as phase B at a flow rate of 0.42 mL/min. The following gradient was applied: 0–12 min: 2–35% B; 12.1–13.5 min: 100% B; and finally 13.6–15 min: to hold 2% B. The injection volume was 5 μL by autosampler on a 2.1 × 100 mm and 1.7 μm BEH C18 column (Waters Corp.; Dublin, Ireland) at 30 °C. The parameters for ESI-QTOF analysis were as follows: capillary temperature 300 °C and voltage 4000 V, drying gas (N_2_) temperature 210 °C with flow 5 L/min, nebulizer pressure 2.0 bar, spectra rate 1 Hz. The mass range was from 100 to 1100 *m*/*z* in negative mode. Data processing was controlled by the software MassLynx 4.0 Application Manager. Identification of polyphenol compounds in the sample was based on PDA and MS spectral data and additionally retention times (R_t_) were compared with those of pure standards if they existed and literature data [15,16]. Hydroxycinnamic acids exhibited an absorbance maximum around 318–325 nm and were expressed as chlorogenic, neochlorogenic and cryptochlorogenic acids, whereas flavonols exhibited an absorbance maximum between 340 and 360 nm and were expressed as quercetin-3-*O*-glucoside and quercetin-3-*O*-rutinoside.

#### 2.3.2. Polymeric Procyanidin Analysis

The phloroglucinolysis method was used for determination content of polymeric procyanidins as proposed previously by Wojdyło et al. [15,17]. For analysis, 5 μL of sample was injected by autosampler on a BEH C18 RP column (2.1 × 5 mm, 1.7 μm; Waters, Milford, MA, USA) at 15 °C. Gradient elution of solvent A (2.5% acetic acid) and solvent B (acetonitrile) had a flow rate of 0.42 mL/min for a duration of 10 min. The detection was recorded at 278 and 360 nm an excitation and emission wavelength, respectively. Quantification of polymeric procyanidins was done using procyanidin B1, (+)-catechin, (−)-epicatechin, and after the phloroglucinol reaction as (+)-catechin and (−)-epicatechin phloroglucinol adduct standards.

#### 2.3.3. Determination of HPLC-PDA-PDA Antioxidant Capacity by an ABTS Online System

A sample for the analysis of ABTS antioxidant capacity online was prepared as described previously by Wojdyło et al. [18]. Antioxidant online profiling analysis was performed at 30 °C using a CADENZA C18 column (75 mm → 4.6 mm i.d., 3 μm; Impact; Tokyo, Japan) where, after the first PDA detector, the mobile phase was mixed with the ABTS radical cation delivered by the additionally pump at a flow rate of 0.5 mL/min. The mixture was reacted on a 25 m long poli(tetrafluoroethylen) (PTFE) reaction coil with 0.25 mm internal diameter. The negative and positive chromatogram obtained at 734 and 280 nm, respectively. The remaining information about chromatographic conditions is described in Section 2.3.1.

### 2.4. In Vitro Pro-Health Potency and Antioxidant Capacity

The lyophilized powdered sample (approx. 1 g for fruits and 0.5 g for leaves) was taken for the determination of biological in vitro activity. The sample was mixed with 7 mL of methanol:water: 37% hydrochloric acid (80:19:1, *v*/*v*/*w*), sonicated for 20 min (Sonic 6D; Polsonic, Warsaw, Poland) and incubated overnight (4 °C). Next, after 24 h, the slurry was centrifuged (MPW-55; Warsaw, Poland) at 19,000× *g* at 4 °C for 10 min to obtain extract for all biological in vitro analysis.

ABTS and FRAP were performed using UV-2401 PC spectrophotometer (Shimadzu; Kyoto, Japan), and the remaining assays were performed using a Synergy H1 microplate reader (BioTek, Winooski, VT, USA). All tests were performed in triplicate.

#### 2.4.1. α-Amylase, α-Glucosidase, and Pancreatic Lipase Inhibitory Activity

The method proposed previously by Wojdyło et al. [19,20] were used for α-amylase, α-glucosidase, and pancreatic lipase inhibitory activity analysis.

The α-amylase and α-glucosidase inhibitory activity is based on a result of reaction of iodide and a β-d-glucosidase, respectively, after enzymatic hydrolysis incubation at 37 °C. The absorbance of α-amylase and α-glucosidase were measured at 600 and 405 nm, respectively. The reference samples contained buffer instead of enzymes and, for positive control, acarbose was used.

The pancreatic lipase inhibitory activity is based on the amount of *p*-nitrophenol formed from *p*-nitrophenyl acetate. Basic samples with enzyme and substrate were incubated at 37 °C, and absorbance was measured at 400 nm. The reference samples contained a buffer instead of enzymes and, for positive control, orlistat was used.

Finally, the results of α-amylase, α-glucosidase, and pancreatic lipase activity are presented as IC_50_ in mg/mL, which means that the sample is able to reduce enzyme activity by 50%.

#### 2.4.2. Cyclooxygenase (COX-1 and COX-2) Assay

Anti-inflammatory activity as COX-1 and COX-2 inhibition by enzyme was determined using a protocol described previously in the COX Inhibitor Screening Assay Kit protocol (Cayman, No. 560131). The results of cyclooxygenase are presented as IC_50_ in mg/mL, which means that the sample is able to reduce enzyme activity by 50%.

#### 2.4.3. Acetylcholinesterase (AChE) and Butylcholinesterase (BChE) Inhibitory Activity

The method proposed a method proposed previously by Wojdyło et al. [19,21] were used for AChE and BChE inhibitory activity. The substrate of acetylcholine iodine and butylcholine chloride is hydrolyzed by the enzyme to thiocholine, which reacts with 5,5′-dithiobis-(2-nitrobenzoic acid) to produce 2-nitrobenzoate-5-mercaptothiocholine and 5-thio-2-nitrobenzoate detected at 405 nm. The reference samples contained a buffer instead of enzymes and, for positive control, galantamine was used. The results are expressed as % inhibition.

#### 2.4.4. FRAP, ABTS^•+^, and ORAC Assay

The method proposed by Benzie & Strain [22]), Re et al. [23], and Ou et al. [24] were used for analysis FRAP (involves determining the ability to reduce Fe^3+^ ions), ABTS (based on measuring the decrease in the color intensity inversely proportional to the antioxidant content) and oxygen radical absorbance capacity (ORAC) (decrease in fluorescence caused by oxidation of a fluorescent substance under the influence of free radicals) assays, respectively.

In addition, 3 mL of reagent TPTZ (2,4,6-Tris(2-pyridyl)-s-triazine) diluted in HCl:FeCl_3_ × 6H_2_O:acetate buffer at pH 3.6; (1:1:10, *v*/*v*/*v*) was mixed with 1 mL of sample extract. After 10 min of reaction, the absorption at 593 nm was measured.

In addition, 3 mL of ABTS (2,2′-azine-bis-(3-ethylene-benzothiazoline-6-sulfonic acid) were mixed with 0.03 mL of sample extract. After 6 min of reaction, the absorption at 734 nm was measured.

2,2′-Azobis(2-amidinopropane)dihydrochloride was mixed with a sample extract; then, phosphate buffer and fluorescein (incubated at 37 °C) were added and measured every 5 min at an excitation and an emission wavelength of 493 and 515 nm, respectively, during 50 min. The blank was a phosphate buffer.

Trolox concentrations 0.050 to 0.900 mM and 0.100 to 0.900 mM were used for the calibration curve (R^2^ = 0.9950) for calculated FRAP and ABTS^•+^, respectively.

Trolox solutions (12.5, 25.0, 50.0, and 75.0 μM) were used for the calibration curve and finally results were obtained by comparing the area under the fluorescence decrease curves over time with the area. The FRAP, ABTS, and ORAC results were expressed in mmol TE (Trolox)/100 g sample dw.

### 2.5. Statistical Analysis

For each cultivars, three samples were analyzed with *n* = 3 repetition and the final results are present as mean value with the standard deviation (SD) in the tables. One-way analysis of variance (ANOVA) followed by Tukey’s HDS test at *p* < 0.05. Linear dependence was calculated by Pearson’s correlation (r) and the multivariate analysis was performed by applying principal components analysis (PCA). Software *XLSTAT* 2017 (Addinsoft, New York, NY, USA) was used for all statistical analysis.

## 3. Results and Discussion

### 3.1. Phytochemicals Constituents Profile of Apricot Leaves Extract

#### 3.1.1. Identification of Polyphenolic Compounds

Phytochemical constituent profiles of *Prunus armeniaca* L. leaf extract were subsequently tentatively identified by liquid chromatography electrospray ionization-quadrupole-time of flight mass/mass spectrometry (LC-ESI-QTOF-MS/MS) in negative ion electrospray mass spectra analysis. The retention time (R_t_), molecular formula and ion, ions after defragmentations, and wavelengths (λ_max_) of maximum absorption in the visible region are shown in Table 1. Finally, a total of fifteen phytochemicals were detected in PrALe, eleven of which were hydroxycinnamic acid derivatives, and four flavonol derivatives.

All compounds with the molecular formula C_16_H_18_O_9_ had similar absorbance spectra with λ_max_ at 325 nm and also had the same negatively charged molecular ion at *m*/*z* 353.22. MS/MS fragmentation presents basic mass spectra as 191.23 and other *m*/*z* as: 179.01, 161.20 and 135.22. Additionally, these peaks were co-chromatographed with the authentic standards and identified as 3-, 4-, 5-*O*-caffeoylquinic acid. The last compound with the formula C_16_H_18_O_9_ at R_t_ = 9.19 min was identified as *cis*-5-*O-*caffeoylquinic acid. Recently, Jaiswal et al. [8,25] in a previous paper reported the hierarchical fragmentation scheme of similar molecules. In addition, a previous report showed the accumulation of different caffeoylquinic acid compounds in *Lonicera* [25], in *Persea americana* [26], in mulberry (genus Morus) [27], in blueberry [28], or in chokeberry [4] leaves. Caffeoylquinic acid derivatives are also typical components for fruits such as apricot [29] or quince [30].

Two compounds with the formula C_16_H_18_O_8_ were identified as 3- and 5-*O*-*p*-coumaroyl-quinic acid due to the base peak at *m*/*z* 337.23, accompanied by a dominant fragment at *m*/*z* 163.22 corresponding to [*p*-coumaric acid–H]^−^ and 191.23 [quinate]^−^ as previously reported [8,25]. The MS/MS fragment of *m*/*z* 337.23 at Rt 9.19 min gave a base signal at *m*/*z* 191.23 and a low intensity ion at *m*/*z* 163.22, which is characteristic for 5-*O*-*p*-coumaroyl-quinic acid. The identification of these molecules was also found to be consistent with published data [31].

Three compounds at Rt 6.15, 7.90, and 8.64 min after fragmentation give C_6_H_10_O_5_ based on the signal at *m*/*z* 162.22 suggesting the presence of a hexosyl residue such as glucoside. As for the compound with C_15_H_18_O_9_ at Rt 6.15 min, the molecular weight was at *m*/*z* 341.22 via the loss of a *m*/*z* 135.22 ([caffeic acid–H]^−^) and after loss of the sugar moiety *m*/*z* 179.01. This compound was identified as caffeoyl-glucoside.

The observation of loss of a hexosyl moiety from molecular formula C_15_H_17_O_8_ presents the main peak at *m*/*z* 163.22 ([*p*-coumaric acid–H]^−^) and the presence of the parent ion at *m*/*z* 325.23, which allows this compound to be identified as *p*-coumaroyl-glucoside.

The compound with C_16_H_20_O_9_ at Rt = 8.64 with the base peak at *m*/*z* 355.23 via the loss of a *m*/*z* 193.12 ([ferulic acid–H]^−^) also produced a base peak at *m*/*z* 175.21 ([ferulic acid–H–H_2_O]^−^) and *m*/*z* 162.22 ([hexosyl–H]^−^) as a loss of internal sugar fragmentations suggested the presence of a feruloylglucoside moiety [25]. These compounds were previously mentioned for leaves of different berry leaves [32].

Two compounds present the molecular formula C_17_H_20_O_9_ with the same pseudomolecular ion at *m*/*z* 367.23. Compared to literature data, these compounds are 3- and 4-*O*-feruloylquinic acids. The two positional isomers were identified by their distinct fragmentations where 3-*O*-feruloylquinic acid yielded an intense MS/MS ion at *m*/*z* 193 [ferulate], while 4-*O*-feruloylquinic acid gave an abundant MS/MS ion at *m*/*z* 173.22 ion and weak ions at *m*/*z* 193.21 and 191.23. By comparison with the literature, feruloylquinic acid derivatives were previously detected in *Zanthoxylum* [33] and *Lonicera* [25] leaves but not in chokeberry leaves [4].

Three different quercetin and one kaempferol derivatives were detected corresponding to spectral data. As previously described [26] in quercetin or kaempferol derivative compounds, a major fragmentation profile occurs which corresponds to an -*O*-glycosidic cleavage.

At Rt 11.60 and 11.77 min, compounds with the same molecular mass of *m*/*z* 609.14 with the molecular formula C_27_H_30_O_16_ were eluted as reported by Pascoal et al. [6]. Both presented the same spectrum and showed the majority fragment after defragmentation at *m*/*z* 301.18, corresponding to the loss of a aglycone fragment—quercetin. Therefore, the compound at Rt 11.77 min co-eluted with standards was identified as quercetin-3-*O*-rutinoside and the compound at Rt 11.60 as its isomer.

The third quercetin derivatives with the molecular formula C_21_H_20_O_12_ displayed an ion at *m*/*z* 463.17 where, after suffering, the neutral loss of *m*/*z* 162 showed an aglycone at *m*/*z* 301.18, which corresponds to the characteristic aglycone moiety attributed to quercetin [6]. These data suggest that the compound could be identified as quercetin-3-*O*-glucoside, which was compared to the spectral and mass data for the standard compound. Similar to the present data, these compounds were found previously for kenaf [6], chokeberry [4], and blueberry [28] as well as other berry [32] leaves.

The compound with the molecular formula C_27_H_30_O_11_ at Rt 13.03 min exhibited a deprotonated molecular ion at *m*/*z* 593.19, with the loss of *m*/*z* 308.11 ([rutinoside–H]^−^) and finally presented a characteristic aglycone fragment at *m*/*z* 285.19, which was attributed to kaempferol. Thus, in some literature data [27], this compound was characterized as kaempferol-3-*O*-rutinoside. Kaempferol-3-*O*-rutinoside is mostly a characteristic compound for *Lonicera henryi* L. (Caprifoliaceae) leaves [25], kenaf [6] leaves, or berries and their leaves [32].

#### 3.1.2. Quantification of Polyphenolic Compounds

The total content of polyphenolic compounds of PrALe calculated as the sum of individual compounds is shown in Table 1. The PrALe of the Early orange cultivar displayed the highest content while the Somo cultivar presented the lowest content. The main detected polyphenolic fractions were hydroxycinnamic acids (45–80% tPc) > flavonols (8–48%) >> polymeric procyanidins (7–13%). The Early orange cultivar exhibited the highest content of hydroxycinnamic acids (406.1 g/kg dw) and polymeric procyanidins (60.8 g/kg dw), while the Somo cultivar presented equal hydroxycinnamic acid and flavonol contents (223.5 and 235.4 mg/kg dw) and only 33.4 g/kg dw polymeric procyanidins. Hydroxycinnamic acid content was predominant too for the Harcot cultivar, and polymeric procyanidins were the highest among all investigated apricot leaf extracts.

According to Campbell et al. [29], the total polyphenols in apricot fruits ranged from 44.0 to 345.1 mg/100 g dw. Carbone et al. [9] mentioned that total polyphenol content was maximal for different apricot cultivars as 1420 mg gallic acid equivalents (GAE)/100 g dw. High concentrations of tPc in apple leaves [30], sour cherry leaves [16], and chokeberry leaves (22.2 g GAE/kg dw) [4] were noted in the literature. In the leaves of Saskatoon, the main fraction consisted of quercetin glycosides, hydroxycinnamic acids, and flavan-3-ols (41; 36; 23%, respectively) [34]. Campbell et al. [29] mentioned that apricot fruits are richer in carotenoid compounds than phenolics. A previous paper reported that *Prunus armeniaca* L. leaves were characterized by the highest content of chlorophylls and lower content of carotenoids compared to other fruit tree leaves, i.e., apple, quince, pear, peach, plums, and sour and sweet cherries [12].

The dominant hydroxycinnamic acids were caffeoylquinic acid derivatives, especially 5-*O*-caffeoylquinic acid (chlorogenic acid), which was consistent with the previous findings [29] for apricot fruits. Early orange and Harcot cultivars accumulated the greatest amounts of 5-*O*-caffeoylquinic acid (317.0 and 216.4 g/kg dw), and, in the Somo cultivar, their content was the lowest. The rest of hydroxycinnamic acids, besides 3-*O*-caffeoylquinic acid (neochlorogenic acid), showed lower content than 16 g/kg dw. Likewise, it is very important to communication that members of this major class of hydroxycinnamic acids not only have a nutraceutical impact on the apricot fruit quality but also have a technological impact. 5-*O*-Caffeoylquinic acid is well known as a principal substrate of polyphenol oxidase activity, which catalyzes the oxidation of di-phenols to *o*-quinones, leading to brown pigments when processing apricot fruits for various products [9].

Flavonols were not particularly abundant in the Early orange cultivar (41.3 g/kg dw), in contrast to Somo and Harcot cultivars (235.4 and 238.0 g/kg dw). The dominant flavonol was quercetin-3-*O*-rutinoside, which in the Somo cultivar was the highest (222.1 g/kg dw), but Early orange accumulated the lowest content (<36 g/kg dw). The other flavonols had lower content than 22 g/kg dw. These results showed that PrALe still is abundant in flavonols compared to other leaves such as *Pistacia* [7] or chokeberry [4]. High content of flavonols reflects the physiological function in response to high solar radiation [7], which gives a promising therapeutic effect.

Flavanols such as monomers and dimers and polymeric proanthocyanins which were minor peaks with the PDA detector operating at 280 nm were quantified by UPLC with the fluorescence detector by the phloroglucinolysis methods. Higher concentrations of polymeric procyanidins in PrALe were found in the Harcot cultivar (83.5 g/kg dw) than in the rest of the analyzed cultivars. Phloroglucinol products of PrALe indicated that phenolics in PrALe consist of polymers of (−)-epicatechin and a small amount of (+)-catechin as terminal units. The degree of polymerization was up to 3, which means that the flavan-3-ol fraction consists of monomers, dimers, or trimers rather than polymeric procyanidins. Some selected cultivars of pear leaves showed a significantly lower total concentration of flavanols, maximally 10–11 g/kg dw [14] and apple 17–25 g/kg dw [17], than analyzed PrALe.

There are descriptions showing that cultivar has a meaningful influence on the profile of phenolic compounds on PrALe, which is mentioned in the literature [9,26,34]. Plants require polyphenols for coloring, growth, reproduction, resistance to pathogens and for many other functions [26]. It is well recognized that the phytochemical composition of plant extracts hinge on genetic composition, plant species, cultivar, phenological stage, geographical location and environmental conditions, biotic and abiotic stressors such as temperature, water availability (precipitation or drought), sunlight intensity, and many others. Since the plants cannot escape from their biotic and abiotic stressors, leaves are the first barrier for safety of fruits, protecting them against these factors [14,35].

### 3.2. In Vitro Pro-Health Potency and Antioxidant Capacity

Due to the fact that the biological activity is multifaceted, it should be evaluated by several different methods. For this reason, in the present study, biological activity was evaluated as antioxidant capacity (ABTS online method, ORAC, ABTS, FRAP) and in vitro enzymatically based methods, i.e., α-amylase, α-glucosidase and pancreatic lipase, acetylcholinesterase and butylcholinesterase (AChE and BChE) inhibitory activity and cyclooxygenase (COX-1 and COX-2) assay. All results are present in Table 2.

#### 3.2.1. Antioxidant Capacity and Online Antioxidant Potential

A statistically significant effect (*p* < 0.05) on the antioxidant capacity was present (Table 2). The ranking of the ABTS^•+^ and ORAC capacity (mmol Trolox/100 g dw, *p* < 0.05) varied from 167.8 to 72.7, and 411.8 to 297.5, respectively, and it was in the following order: Somo > Early orange > Harcot cultivars. In FRAP assay, the activity of the leaf extracts was in the order (mmol Trolox/100 g dw): Somo (71.4 mmol) > Harcot (50.4 mmol) >> Early orange (17.1 mmol). Generally, PrALe of the Somo cultivar presented the highest scavenging activity among all tested extracts from apricot leaves. Finally, it is known that antioxidant capacity is associated with the contents of phenolic compounds but corresponds not only with a high amount of phenolic compounds as basically bioactive compounds in plant extracts but also on the content of each compound, as indicated by the online antioxidant activity analysis. The principal antioxidant power of the Somo cultivar extract was represented by quercetin-3-*O*-rutinoside, which was dominant in this extract, but the Harcot cultivar extract was rich in caffeoylquinic acid derivatives.

**Figure 1:**
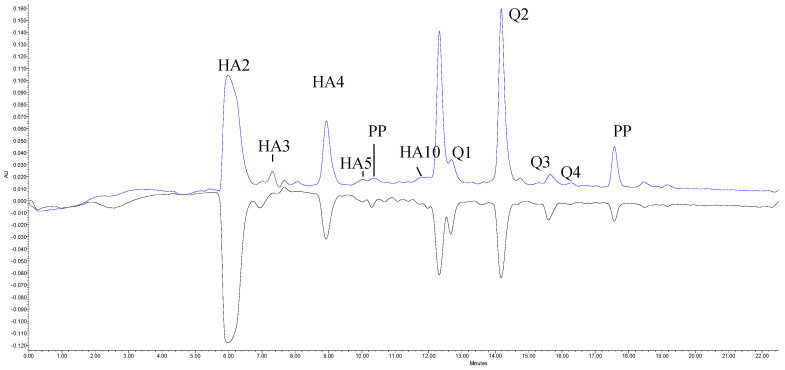
HPLC-PDA traces at 280 nm and on-line antioxidant detection (734 nm) basic polyphenols component of PrALe of Harcot cv. HA2-4-*O*-caffeoylquinic acid; HA3-3-*p*-coumaroyl-quinic acid; HA4-5-*O*-caffeoylquinic acid; HA5-*p*-coumaroyl-glucoside; HA8-*cis*-5-*O*-caffeoylquinic acid; HA10-4-*O*-feruloylquinic acid; Q1-quercetin-3-*O*-rutionoside; Q2-quercetin-3-*O*-rutinoside; Q3-quercetin-3-*O*-galactoside; Q4-keampferol-3-*O*-rutinoside; PP-polymeric procyanidins.

To better recognize which compounds of PrALe were answerable for the antioxidant activity, UPLC-PDA-PDA online analysis with ABTS radicals was performed. The area of negative peaks reordered on the lower chromatogram at 734 nm conform to the activity of individual phenolic compounds of PrALe after reaction with ABTS radicals. Figure 1 presents three main compounds that contribute to antioxidant power, which were the present order: quercetin-3-*O*-rutinoside (Q2 and Q1) > derivatives of caffeoylquinic acid (HA2, HA4, and HA8) > polymeric procyanidins (PP) >> remaining minor quantified compounds, i.e., hydroxycinnamic acid (HA3, HA5, HA7), flavonols (Q3 and Q4) or flavan-3-ols. 

The powerful antioxidant properties of polyphenolic compounds are closely related to the structure [36]. The compound quercetin present in ring B a catechol structure, and in ring C a 2,3-double bond, allowing for delocalization of the phenoxyl radical electron to the flavonoid nucleus and high anti-radical effectiveness, while the 3,4-position of dihydroxylation on the phenolic ring in caffeic acid shows increased antioxidant activity as compared to *p*-coumaric or ferulic acids and their derivatives or other hydroxycinnamic acids [36]. Additionally, flavan-3-ols display powerful antioxidative properties, but how the online chromatogram of PrALe additionally presents the amount of individual compounds is important. Our findings are similar to those published in the literature [37].

#### 3.2.2. α-Amylase, α-Glucosidase, and Pancreatic Lipase Inhibitory Activity

Obesity is a global public health concern that has been described as a risk factor for serious diseases affecting a significant part of the population worldwide, i.e., type II diabetes, hypertension, cardiovascular, and some forms of cancer [4,38,39].

PrALe was analyzed for its ability to inhibit α-amylase, α-glucosidase, and pancreatic lipase. All PrALe tested presented some inhibition of the tested enzyme, but some differences were noted between them in effectiveness. PrALe was a more effective pancreatic lipase and α-glucosidase inhibitor than α-amylase. Consequently, it was noted that α-amylase was less readily inhibited by PrALe, and there was no significant difference in pancreatic lipase activity between the cultivars. The extent of inhibition of α-glucosidase was related to *p*-coumaroyl-quinic acid (Person correlation; r = 0.913) and quercetin-3-*O*-rutinoside (Person correlation; r = 0.754) content, where for pancreatic lipase the inhibition related to all caffeoylquinic acid derivatives (r > 0.998) and some flavonols such as quercetion-3-*O*-rutinoside and -3-*O*-galactoside and kaempferol-3-*O*-rutinoside (r > 0.985) was decisive for activity. The most effective fraction of PrALe in inhibiting α-amylase contained appreciable amounts of polymeric procyanidins (r = 0.918) and some phenolic acid (r > 0.755). This result intensely implies that inhibition of α-amylase, α-glucosidase, and pancreatic lipase is affected by different phenolic components or their fractions.

It has long been demanded that polyphenolic fractions isolated from plants can change glucose utilization in mammals, causing insulin and anti-obesity effects [34]. The inhibition of amylase by green tea, raspberry, strawberry, grape, or cocoa extracts was attributed to polymeric procyanidin components as reported by Lavola et al. [34]. It has also been acknowledged that proanthocyanidins (tannins) and ellagic acid derivatives isolated from Banaba (*Lagerstroemia speciosa* L.) leaves present some inhibitors against α-amylase. In addition, research by Zang et al. [39] showed that caffeoylation of -OH groups in quinic acid and quercetin-3-*O*-glucosidase was important for the α-glucosidase inhibitory activity. Martinez-Gonzalez et al. [38] reported a higher pancreatic lipase inhibitory potency for quercetin than other phenolic compounds, which is in agreement with the present results.

#### 3.2.3. Cyclooxygenase (COX-1 and COX-2) Assay

Cyclooxygenase (COX-1 and COX-2) is an enzyme producing prostaglandins (PGs) which are involved in inflammatory processes of several different disorders and diseases such as diabetes, cancer (e.g., those of the breast colon, and lung) or atherosclerosis [40]. In the past period, people were focused on health benefits of secondary metabolites to treat inflammatory complaints, but it is still important to find new plant sources rich in phenolic compounds. The COX-1 inhibition by PrALe was at a similar level, the IC_50_ value ranging between 0.9 and 2.2, but the COX-2 inhibition had an IC_50_ range of 1.8–9.7. PrALe of the Early orange and Harcot cultivars presented higher COX-1 activity than the Somo cultivar. However, PrALe of the Somo and Harcot cultivars presented higher potency to inhibit cyclooxygenase-2 than PrALe of the Early orange cultivar. Additionally, the results of the COX inhibition assay showed that COX-2 was generally less susceptible to inhibition by PrALe. Similar effects were observed by Hong et al. [40] for the inhibition assay by leaves of green and black tea polyphenols.

The varied anti-inflammatory potential of PrALe can be calculated by the Pearson’s correlation and description between its phenolic compounds and the effects: the only positive correlation was found between COX-1 and *p*-coumaroyl-glucoside acids (r = 0.997), while COX-2 positively correlated with caffeoyl-glucoside, 4-*O*-caffeoylquinic acid, 3-*p*-coumaroyl-quinic acid, 5-*O*-caffeoylquinic acid, feruloyl-glucoside content (r > 0.912). The results indicated that the presence of flavonoids negatively correlated with cyclooxygenase inhibition, and probably the presence of a large polar conjugated sugar moiety. A similar relationship was presented in literature data [41]. The specific inhibitory effect and the structural association of PrALe on cyclooxygenase (both COX-1 and COX-2) need to be examined further.

#### 3.2.4. Acetylcholinesterase (AChE) and Butylcholinesterase (BChE) Inhibitory Activity

Finding some natural origin inhibitors of AChE and BChE, considered to be promising therapeutic agents for the treatment of neurological disorders such as Alzheimer′s and Parkinson′s disease, myasthenia gravis, senile dementia, and ataxia, is extremely important. The inhibitory activities of PrALe towards AChE and BChE are summarized in Table 2. In general, the activity of PrALe was low, less than 10%, but significantly proportional to the polyphenol concentration of evaluated cultivars. PrALe of the Early orange and Harcot cultivars presented higher AChE and BChE activity than the Somo cultivar. There was weak correlation between total polyphenol or flavonol content and both cholinesterase enzymes. A high correlation was noted for AChE and polymeric procyanidins (r = 0.941). It was observed that some caffeoylquinic derivatives of PrALe had a significant role in the inhibition of AChE and BChE (r > 0.712 and r > 0.912, respectively). Despite the fact that AChE and BChE have many structural connections, the dissimilar inhibitory effects of polyphenols are related to minor differences in the structure of the enzymes, e.g., an active site or oxyanion hole. Nevertheless, previous reports on the neuroprotection of polyphenols show a high potential of flavonoids and hydroxycinnamic acids [42]. In light of research by Samaradivakara et al. [43], alkaloids and terpenoids are known to be stronger inhibitors than polyphenolic compounds. Based on the previous results [32], extracts of the leaves of *Macaranga kurzii* were found to inhibit AChE by 40%. Senol et al. [21] evaluated the AChE and BChE inhibitory potential of the extracts from 25 plant species of genera such as *Salvia*, *Hypericum*, *Onosma*, *Thymus*, *Origanum*, *Rosa*, and *Prunus*. The results were presented as an average of AChE inhibition in a similar level range of 12–42% enzyme inhibition.

### 3.3. Principal Component Analysis

**Figure 2:**
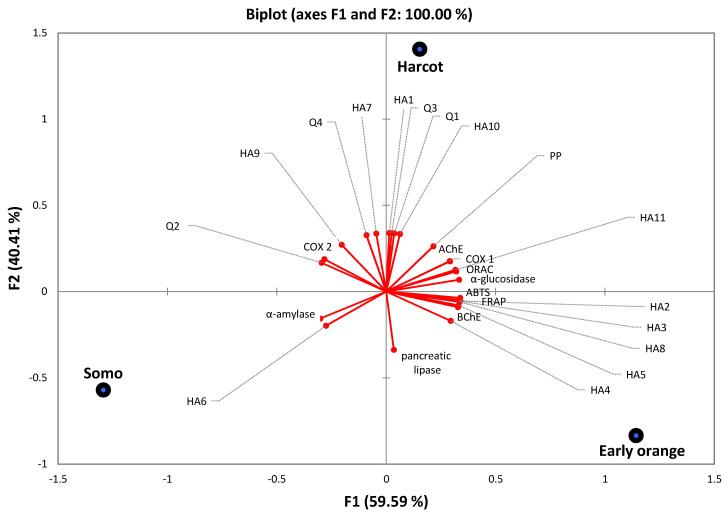
Biplot (PC1 × PC2) of scores and loadings for the PrALe, antioxidant capacity, and enzyme inhibitory. HA1-caffeoyl-glucoside; HA2-4-*O*-caffeoylquinic acid; HA3-3-*p*-coumaroyl-quinic acid; HA4-5-*O*-caffeoylquinic acid; HA5-*p*-coumaroyl-glucoside; HA6-3-*O*-feruloylquinic acid; HA7-feruloyl-glucoside; HA8-*cis*-5-*O*-caffeoylquinic acid; HA9-5-*p*-coumaroylo-quinic acid; HA10-4-*O*-feruloylquinic acid; Q1-quercetin-3-*O*-rutionoside; Q2-quercetin-3-*O*-rutinoside; Q3-quercetin-3-*O*-galactoside; Q4-keampferol-3-*O*-rutinoside; PP-polymeric procyanidins; ORAC-the Oxygen Radical Absorbance Capacity; AChE-acetylcholinesterase; BChE-butylcholinesterase; COX-cyclooxygenase.

Principal components analysis of polyphenolic compounds in leaf extracts of the representative *Prunus armeniaca* L. versus their pro-health action potency was performed, as presented in the biplot in Figure 2. Two principal components, describing 100% of the total data variance, were used for a detailed analysis. Contents of some hydroxycinnamic acids (HA2 = 0.987; HA3 = 0.986; HA4 = 0.866; HA5 = 0.972; HA8 = 0.985, HA11 = 0.929), ABTS, FRAP, ORAC (0.994, 0.990, 0.939), AChE and BChE (0.857 and 0.964), α-glucosidase (0.980), and COX-1 (0.854) strongly correlated with the first component (PC1), described for 59.59%. It was next observed that the second component, describing 40.41% of the variance, strongly positively correlated with contents of quercetins (Q1 = 0.994, Q3 = 0.997, Q4 = 0.964) and the remaining hydroxycinnamic acids (HA1, HA7, HA10 = 0.999–0.983) and moderately positive (0.774) with contents of PP. Similar results for investigated leaves of *Sorbus taxa* were presented by Gaivelyte et al. [44].

Contrary to this, the remaining correlations were negative or weak. According to the biplot, distinct groups were identifiable which were concentrated around three varieties. PrALe of Early orange and Harcot cultivars could be well known among all species as their leaf samples contained great contents of polyphenols positively corresponding with antioxidant capacity (ABTS, FRAP, ORAC) and presented high enzyme inhibition, besides α-amylase, which correlated with PrALe of the Somo cultivar.

## 4. Conclusions

In summary, our results clearly showed that the phytochemical composition and consequently the antioxidant capacity and in vitro enzyme inhibition of the tested samples of polyphenol extracts from *Prunus armeniaca* L. are heterogeneous. The results demonstrate that the differences between extracts are important when finding some new rich sources of bioactive compounds from raw plant materials. Comparison of different polyphenolic extracts of *Prunus armeniaca* L. cultivar leaves according to their quantitative composition revealed that they are exceptional sources of hydroxycinnamic acids. PrALe showed the most effective anti-obesity action via inhibition of pancreatic lipase, cyclooxygenase-1, and antioxidant capacity, especially the oxygen radical absorbance capacity, which was particularly correlated with polyphenolic compounds. Online ABTS radicals clearly indicate that three predominant compounds of PrALe—quercetin-3-*O*-rutinoside > 5-*O*-caffeoylquinic acid > 3-*O*-caffeoylquinic acid—basically contribute to antioxidant potential.

The results of this work suggest that extracts from the *Prunus armeniaca* L. leaves can be applied in different commercial sectors such as food, cosmetics, and pharmaceuticals. On the one hand, the extracts can be used as antioxidants of natural origin that replace synthetic antioxidants in many food and cosmetic applications. On the other hand, they could be considered as a good alternative in the treatment and prevention of diabetes and inflammatory disorders and used in obesity prevention or as an anti-aging agent. Finally, the *Prunus armeniaca* L. leaf extracts could be an important source of interesting molecules for the prevention and treatment of other diseases of the 21st century such as cancer.

## Figures and Tables

**Table 1:** Retention time (R_t_), wavelengths (λ_max_) of maximum absorption in the visible region, mass spectral data, identification, and quantification (mean ± SD) of phenolic compounds of the PrALe by LC-ESI-QTOF-MS/MS.

Tentative Identification	Molecular Formula	R_t_ (min)	λ_max_ (nm)	Molecular Ion [M-H] (*m*/*z*)	Main MS/MS Fragments (*m*/*z*)	Quantification [mg/kg]		
						Somo	Harcot	Early Orange
Hydroxycinnamic acids								
3-*O*-Caffeoylquinic acid	C_16_H_18_O_9_	5.87	324	353.22	191.23	24.27 ± 0.90 b	46.89 ± 1.43 a	22.42 ± 1.11 b
Caffeoyl-glucoside	C_15_H_18_O_9_	6.15	331	341.22	179.01/162.20/135.22	4.50 ± 0.32 c	8.42 ± 0.45 b	13.13 ± 0.12 a
4-O-caffeoylquinic acid	C_16_H_18_O_9_	6.25	331	353.22	191.23/161.20	4.82 ± 0.42 c	9.49 ± 0.52 b	15.19 ± 0.32 a
3-*p*-Coumaroyl-quinic acid	C_16_H_18_O_8_	7.00	311	337.23	163.22	6.65 ± 0.11 bc	7.75 ± 0.73 b	15.92 ± 0.43 a
5-*O*-Caffeoylquinic acid	C_16_H_18_O_9_	7.50	325	353.22	191.23/179.21/135.22	152.71 ± 2.54 c	216.39 ± 2.13 b	316.98 ± 2.49 a
*p*-Coumaroyl-glucoside	C_15_H_17_O_8_	7.90	311	325.23	163.22	11.15 ± 0.67 a	1.26 ± 0.10 c	3.39 ± 0.32 b
3-*O*-Feruloylquinic acid	C_17_H_20_O_9_	7.97	325	367.23	193.21/173.22	3.74 ± 0.12 b	12.12 ± 0.52 a	0.93 ± 0.09 c
Feruloyl-glucoside	C_16_H_20_O_9_	8.64	328	355.23	193.12/175.21/162.20	7.31 ± 0.23 c	9.02 ± 0.23 b	11.19 ± 0.21 a
*cis*-5-*O-*Caffeoylquinic acid	C_16_H_18_O_9_	9.10	314	353.22	191.23/161.20	5.69 ± 0.56 b	7.00 ± 0.56 a	2.60 ± 0.22 c
5-*p*-Coumaroylo-quinic acid	C_16_H_18_O_8_	9.19	315	337.23	191.23/173.22/163.22	2.60 ± 0.22 bc	8.02 ± 0.89 a	3.07 ± 0.16 b
4-*O*-Feruloylquinic acid	C_17_H_20_O_9_	11.10	316	367.23	193.21/191.23/173.22	nd	1.22 ± 0.11 a	1.28 ± 0.19 a
Flavonols								
Quercetin-3-*O*-rutinoside	C_27_H_30_O_16_	11.60	264/355	609.14	301.18	nd	13.31 ± 0.56 a	nd
Quercetin-3-*O*-rutinoside	C_27_H_30_O_16_	11.77	258/354	609.14	301.18	222.11 ± 2.65 a	198.22 ± 1.89 b	35.19 ± 0.51 c
Quercetin-3-*O*-galactoside	C_21_H_20_O_12_	12.25	261/354	463.17	301.18	3.41 ± 0.34 b	4.79 ± 0.13 a	3.35 ± 0.23 b
Kaempferol-3-*O*-rutinoside	C_27_H_30_O_11_	13.03	265/341	593.16	308.11/285.19	9.85 ± 0.67 b	21.70 ± 0.99 a	2.79 ± 0.11 c
Polymeric procyanidin						33.38 ± 1.23 c	83.54 ± 1.54 a	60.83 ± 1.43 b
Degree of polymerisation						2.01	2.63	2.83
Tota polyphenols [g/kg]						492.20 BC	649.13 A	508.27 B

nd—not detected; data are shown as mean (*n* = 3) ± standard deviation; a–c or A–C—in each row, different letters indicates the significant differences at *p* < 0.05 between samples (Tukey’s HSD test).

**Table 2:** Antioxidant capacity (ORAC, FRAP, ABTS; mmol TE/100 g dw), enzymatic in vitro enzyme inhibition of hyperglycemic (α-glucosidase, α-amylase; IC_50_, mg/mL), obesity (pancreatic lipase; IC_50_, mg/mL), cholinesterase (acetylcholinesterase, butylcholinesterase; %), and inflammatory (COX-1 and -2; IC_50_, mg/mL) of PrALe.

Analysis	Polyphenol-Rich Apricot Leaf Extract		
	Somo	Harcot	Early Orange
ABTS	167.83 ± 1.98 a	72.75 ± 2.54 c	131.47 ± 1.45 b
FRAP	71.43 ± 1.00 a	50.41 ± 1.89 b	17.13 ± 1.01 c
ORAC	411.85 ± 2.67 a	297.51 ± 3.76 b	312.57 ± 3.54 b
AChE	5.53 ± 0.41 b	10.33 ± 1.55 a	9.68 ± 1.22 a
BChE	5.93 ± 0.11 bc	6.87 ± 0.25 b	8.55 ± 0.43 a
α-Amylase	3.32 ± 0.05 a	7.52 ± 0.32 b	7.23 ± 0.21 b
α-Glucosidase	3.35 ± 0.6 c	1.30 ± 0.11 b	0.71 ± 0.05 a
Pancreatic lipase	0.17 ± 0.01 a	0.21 ± 0.03 a	0.16 ± 0.01 a
COX 1	2.19 ± 0.21 b	0.74 ± 0.06 a	0.94 ± 0.04 a
COX 2	1.81 ± 0.16 a	2.22 ± 0.03 ab	9.69 ± 0.10 c

ABTS-2,2′-azinobis-(3-ethylbenzothiazoline-6-sulfonic acid) radical cation; FRAP-the Ferric Reducing Ability of Plasma; ORAC-the Oxygen Radical Absorbance Capacity; AChE-acetylcholinesterase; BChE-butylcholinesterase; COX-cyclooxygenase; Data are shown as mean (*n* = 3) ± standard deviation; a–c—in each row, different letters mean significant differences between samples ((Tukey’s HSD test, *p* < 0.05); IC_50_ values correspond to the extract concentration achieving 50% of activity. Data are shown as mean (*n* = 3) ± standard deviation; a–c—in each row, different letters indicate the significant differences at *p* < 0.05 between samples (Tukey’s test).

## Data Availability

Data is contained within the article.
